# Prophylactic antibiotics induce early postcraniotomy seizures in neurosurgery patients: A case series

**DOI:** 10.1097/MD.0000000000031714

**Published:** 2022-11-25

**Authors:** Nawon Lee, Dae-Lim Jee, Hyuckgoo Kim

**Affiliations:** 1 Department of Anesthesiology and Pain Medicine, College of Medicine, Yeungnam University, Daegu, Republic of Korea.

**Keywords:** seizure, antibiotics, cefotiam, neurosurgery

## Abstract

Antibiotics can cause central nervous system disturbances, manifesting as dizziness, confusion, headache, and seizures. Seizures due to antibiotic administration are related to increased excitatory neurotransmission because antibiotics act as competitive antagonists of the γ-aminobutyric acid type A receptor.

**Patient concerns and clinical findings:**

All 5 patients, comprising 4 females and one male and aged 45 to 72 years, underwent open craniotomy with additional surgical maneuvers according to their specific disease. All patients presented American Society of Anesthesiologists Physical Status grades 1 to 2. There were no specific underlying diseases, except hepatitis C and hypertension. However, seizures developed sequentially in the 5 patients after neurosurgery.

**Diagnoses, interventions, and outcomes:**

Early postcraniotomy seizures (PCS) developed in the patients after neurosurgery. Prophylactic antibiotics were administered in all cases to prevent infection due to open craniotomy. This included the administration of 10 g and 2 g of an antibiotic (cefotiam HCL; Jetiam Intravenous Injection 1g^®^) an hour before the surgery in the ward and half an hour before the surgery in the operating room, respectively. After surgery, cefotiam HCL 2 g was administered in all patients on the day of surgery. Five patients had myoclonic seizure or generalized tonic–clonic seizure several times at emergence or in the intensive care unit.

**Lessons:**

Early PCS occurred in every patient when an overdose of the prophylactic antibiotic was administered. This report showed that the preoperative prophylactic antibiotic cefotiam administered in double doses evoked early PCS within a few hours of drug administration. Furthermore, such experiences caution that preoperative intravenous cephalosporins, including cefotiam, should be administered carefully in open craniotomy.

## 1. Introduction

Antibiotics have been essentially administered in patients undergoing surgery to prevent infective complications. However, physicians are always concerned about the administration of antibiotics because they can cause adverse effects, including hypersensitivity, coagulation abnormalities, central nervous system (CNS) disturbances, hepatotoxicity, and gastrointestinal disturbances. CNS disturbances manifest as dizziness, confusion, headaches, and seizures. Particularly, seizures provoked by antibiotic administration are related to increased excitatory neurotransmission, because the antibiotics act as competitive antagonists of the γ-aminobutyric acid type A (GABAA) receptor.^[1]^ The actual incidence of seizures due to antibiotics is unknown, epidemiological data are lacking, and evidence of an association between seizures and antibiotics is very low. Moreover, accidental administration of an overdose of antibiotics in humans has rarely been reported.

We encountered 5 cases of early postcraniotomy seizures (PCSs) that developed after neurosurgery. In all 5 cases, the prophylactic antibiotic cefotiam was administered intravenously during the preoperative period. Antibiotic-related symptomatic seizures have mainly been reported in association with β-lactams. Cefotiam, a second-generation cephalosporin developed in Japan, exhibits broad-spectrum activity against gram-positive and gram-negative bacteria. There is no published evidence specifically related to cefotiam in humans, although a few case reports have been published. We describe the clinical course of 5 patients who inadvertently received a massive dose of antibiotics intravenously.

## 2. Case report

We encountered 5 cases of postoperative seizures after neurosurgery, and the seizures developed sequentially. They were 1 male and 4 female patients, aged 45 to 72 years, who presented American Society of Anesthesiologists Physical Status grades 1 to 2. There were no specific underlying diseases, except hepatitis C and hypertension. All the patients underwent open craniotomy with additional surgical maneuvers according to their specific disease. The blood test, chest radiography, and electrocardiography findings of all the patients were unremarkable. Their vital signs were within the normal range, and neurological examinations revealed no abnormalities (Table 1). Before surgery, glycopyrrolate 0.2 mg was injected intravenously as premedication. Routine monitoring, including blood pressure measurement, electrocardiography, and pulse oximetry, was performed in the operating room (OR). For general anesthesia induction, propofol and remifentanil were administered using a target-controlled infusion pump. Only one patient was anesthetized with a bolus of propofol and sevoflurane inhalation for induction and maintenance of general anesthesia (Table 2). Rocuronium was administered for muscle relaxation in all cases. During general anesthesia induction, no adverse events due to the anesthetic agents were reported. At the end of surgery, pyridostigmine 10 mg and glycopyrrolate 0.4 mg were injected intravenously in all the patients. In all cases, a prophylactic antibiotic was administered to prevent infection due to open craniotomy. This included administration of 10 g and 2 g of an antibiotic (cefotiam HCL; Jetiam Intravenous Injection 1g^®^) an hour before the surgery in the ward and half an hour before the surgery in the OR, respectively. Furthermore, cefotiam HCL 2 g was administered after the surgery in all patients on the day of surgery.

**Table 1:** Preoperative findings of the 5 patient.

	First patient	Second patient	Third patient	Fourth patient	Fifth patient
Age/sex	45/F	60/F	49/F	66/F	72/F
Diagnosis	Unruptured aneurysm on Lt. MCA bifurcation	Meningioma on anterior clinoid process	Lt. facial spasm	Unruptured aneurysm on Lt. MCA bifurcation	Unruptured aneurysm on ACA
Underlying disease	None	HCV carrier	None	None	HTN
Preoperative evaluation	NS	NS	NS	NS	NS
Neurologic exam	Intact	Intact	Intact except facial spasm	Intact	Intact

F, female; M, male; Lt., left; MCA, middle cerebral artery; ACA, anterior cerebral artery; HCV, hepatitis C virus; HTN, hypertension; NS, nonsignificant.

**Table 2:** Perioperative course of the 5 patients.

	First patient	Second patient	Third patient	Fourth patient	Fifth patient
Induction and maintenance	TIVA	TIVA	Propofol + sevoflurane	TIVA	TIVA
Intraoperative V/S	WNL	WNL	WNL	WNL	WNL
Transfusion	-	-	-	-	Packed RBC 2
Seizures at emergence	No seizure	Myoclonic seizure	No seizure	Myoclonic seizure	Tonic–clonic seizure
Seizure at NICU	Tonic–clonic seizure for several times	Myoclonic seizure for several times	Myoclonic seizure for 2 times	Myoclonic seizure for several times	Tonic–clonic seizure for several times
Treatment	Lorazepam valproate sodium infusion	Lorazepam thiopental sodium infusion	Lorazepam valproate sodium infusion	Lorazepam thiopental sodium infusion	Lorazepam valproate sodium infusion
Progress	Day 1: alert and neurological state intact	Day 1: drowsy and neurological state intact	Day 1: alert and neurological state intact	Day 3: drowsy and neurological state intact	Day 5: both lung pneumonia Day 49: Expire d/t Sepsis

NICU = neurointensive care unit; d/t, due to, RBC = red blood cells, TIVA = total intravenous anesthesia; WNL = within normal limits.

The first patient was a 45-year-old female diagnosed with an unruptured aneurysm at the left middle cerebral artery bifurcation. She underwent left pterional osteoplastic cranioplastic craniotomy and clipping of the aneurysmal neck under general anesthesia for 145 minutes. At the end of surgery, the patient was able to breathe by herself, and the tracheal tube was removed. Although she presented a postoperative mental state of stupor, her vital signs were stable. In the intensive care unit (ICU), the patient had generalized tonic–clonic seizures several times on the day of surgery. The patient’s laboratory findings were nonspecific in the OR and ICU (Table 3). The next day, her mental state changed to alert, and the neurological status was intact. The patient was discharged 10 days postoperatively.

**Table 3:** Arterial blood gas analysis in the operating room and neurointensive care unit.

Patient		pH	PaCO_2_ mmHg	PaO_2_ mmHg	BE mmol/L	HCO_3_ mmpl/L	SaO_2_ %	Na^ + ^mEq/L	K^ + ^mEq/L	Ca^2+^ mEq/L
1	OR	7.46	35.0	258	1.2	24.9	100	144	3.5	4.36
	NICU	7.44	37.8	114	1.5	25.1	97.4	145	3.8	4.29
2	OR	7.46	31	270	−1.2	22.0	100	140	3.0	4.00
	NICU	7.45	32.7	151	−1.0	22.3	98.8	146	2.6	4.49
3	NICU	7.32	36.4	147	−6.8	18.2	98.5	144	3.8	4.24
4	NICU	7.43	38.7	75.1	1.7	25.4	97.9	142	2.9	4.19
5	OR	7.38	38	247	−2.4	22.5	100	131	5.0	4.20
	NICU	7.44	39.9	79.2	2.6	27.7	94.8	136	4.8	4.24

BE = base excess, NICU = neurointensive care unit, OR = operating room, PaCO_2_ = partial pressure of carbon dioxide, PaO_2_ = partial pressure of oxygen, SaO_2_ = oxygen saturation in the arterial blood.

The second patient was a 60-year-old female diagnosed with a meningioma in the anterior clinoid process. She underwent right pterional craniotomy and gross total removal of the tumor mass with a navigation system under general anesthesia for 195 minutes. During emergence, the patient had a myoclonic seizure; hence, she was transferred to the ICU in the intubated state. Although she presented a postoperative mental state of stupor, her vital signs were stable. In the ICU, the patient had myoclonic seizures several times on the day of surgery. The patient’s laboratory findings were nonspecific in the OR and ICU (Table 3). The next day, her mental state changed to drowsy, and the neurological status was intact; hence, she was successfully extubated. The patient was discharged 12 days postoperatively.

The third patient was a 49-year-old female with a chief complaint of left facial spasm. Left retromastoid approach of osteoplastic craniotomy and microvascular decompression of the seventh nerve were performed under general anesthesia for 180 minutes. At the end of surgery, the patient was able to breathe by herself, and the tracheal tube was removed. Although her postoperative mental state was deep drowsy, her vital signs were stable. In the ICU, the patient had myoclonic seizures twice on the day of surgery. The initial arterial blood gas analysis in the ICU revealed slight acidic arterial blood with adequate oxygenation (Table 3). The next day, her mental status changed to alert, and the neurological status was intact. The patient was discharged 7 days postoperatively.

The fourth patient was a 66-year-old female diagnosed with an unruptured aneurysm at the left middle cerebral artery bifurcation. She underwent left pterional osteoplastic craniotomy and clipping of the aneurysm under general anesthesia for 150 minutes. During emergence, the patient had a myoclonic seizure; therefore, she was transferred to the ICU in an intubated state. She presented a postoperative mental state of stupor, and her blood pressure was very high. In the ICU, the patient had myoclonic seizures several times on the day of surgery. The initial arterial blood gas analysis in the ICU showed normal results with adequate oxygenation (Table 3). On day 3 postoperatively, her mental state changed to drowsy, and the neurological status was intact; hence, she was successfully extubated. The patient was discharged 12 days postoperatively.

The fifth patient was a 72-year-old male diagnosed with an unruptured aneurysm of the anterior communicating artery. The patient underwent left pterional osteoplastic craniotomy and clipping of the aneurysm under general anesthesia for 265 minutes. During emergence, the patient had a generalized tonic–clonic seizure; hence, he was transferred to the ICU in the intubated state. Although he presented a postoperative mental state of stupor, his vital signs were stable. In the ICU, the patient had generalized tonic–clonic seizures several times on the day of surgery. The patient’s laboratory findings were nonspecific in the OR and ICU (Table 3). On day 5 postoperatively, chest computed tomography revealed pneumonia in both the lungs, and he had fever for several days. The creatinine level remained high (3.22 mg/dL). On day 49 postoperatively, the patient expired due to sepsis and acute renal failure.

Lorazepam 2 mg was intravenously injected each time to stop seizures. However, the seizures were not controlled well, and thiopental sodium 6 g was administered. In the last case, the patient had a generalized tonic–clonic seizure during emergence. Therefore, thiopental sodium 500 mg was injected, and the patient was transferred to a neuro-ICU in the intubated state (Table 2). All patients’ seizures ceased on the second day after surgery.

## 3. Discussion

Neurosurgical patients under general anesthesia have a number of etiologic factors for developing seizures, such as cerebrovascular disease, hypotensive syndrome, metabolic disorder, and even general anesthesia itself.^[2]^ In addition, neurosurgical procedures can cause complications such as brain edema, postoperative hemorrhage, cerebral ischemia, cranial nerve dysfunction, and seizures.^[2]^ Clinical seizures and epileptiform discharges on electroencephalogram are observed in 25% of the patients in the acute postoperative period after craniotomy. Although the exact incidence of perioperative seizures in vascular neurosurgery is unknown, the reported incidence of seizures from observational studies is in the range of 4 to 42%.^[3]^ In another report, the incidence of seizures after supratentorial surgery related to nontraumatic pathology is estimated as 15 to 20%.^[4]^ The incidence of seizures in patients who undergo craniotomy is 4.9 to 21.2% for ruptured cerebral aneurysms and 0.1 to 4.4% for unruptured cerebral aneurysms.^[5]^ However, it is difficult to prove the correlation between the causative pathology and abrupt development of seizures. In our case series, all 5 neurosurgical patients who underwent open craniotomy experienced early PCS several times sequentially. Three of the 5 patients experienced seizures during emergence from general anesthesia. Early PCS usually includes seizures occurring in the first 24 h postoperatively as craniotomy-related seizures. Chung et al warned that intraoperative cefazolin irrigation increased the risk of early PCS in patients undergoing craniotomy, especially during clipping of unruptured aneurysms.^[6]^ They included seizures occurring in the first 24 h postoperatively as the early PCS. Michenfelder et al specified that early PCS is the occurrence of seizures in the first 6 h postoperatively and performed a retrospective comparison of the incidence of early PCS.^[7]^ Although seizures frequently occur in neurosurgical patients, it is very unusual that early PCS sequentially developed in all 5 cases. In these cases, postoperative hematologic and radiologic evaluations revealed normal findings. Serum sodium, calcium, and glucose levels, which can provoke both abnormal neuronal depolarization and immediate disruption of ionic balance, were within normal limits, and all patients had stable vital signs. According to several reports, the PCS induced by pathology or surgical manipulation usually develops within the first or second week after craniotomy.^[3–5]^ There are differences in the onset time between PCS induced by pathology or surgical manipulation and that in our 5 cases.

In these cases, the prophylactic antibiotic drug cefotiam was administered before surgery. The drug has been newly introduced to replace the existing prophylactic antibiotics. It is recommended that the dose of cefotiam should be up to 6 g daily for adults. However, 10 g and 2 g of cefotiam were administered in the preoperative and intraoperative periods, respectively. We focused on our clinical suspicion that the antibiotic drug administration caused the seizures. It is well known that antibiotics have neurotoxic effects during the perioperative period, although they are often accompanied by other predisposing factors. Notably, more than half the reports on neurotoxicity, including those on seizures, have shown an association with cephalosporins and other β-lactams.^[8]^ Cefotiam is a second-generation cephalosporin developed in Japan and has broad-spectrum activity against gram-positive and gram-negative bacteria. It can cause nausea and vomiting, diarrhea, hypersensitivity reactions, nephrotoxicity, and hepatic dysfunction by oral intake or intravenous injection.^[9,10]^ Similar to other cephalosporins, high concentrations of cefotiam in the brain have been linked to CNS toxicity. It decreases the seizure threshold by acting as a competitive antagonist of the GABAA receptor and increases excitatory neurotransmission.^[1]^ Consequently, cefotiam induces serious adverse effects in the CNS, such as confusion, twitching, and seizures. There is a case report of generalized myoclonic seizure due to accidental intrathecal infusion of cefotiam.^[11]^ In this report, the seizures developed 2 h after the administration, and the measured value of the drug in the cerebrospinal fluid (CSF) increased significantly. In all our cases, the double dose of the prophylactic antibiotic cefotiam provoked seizures within 6 h after preoperative administration. These results showed that cefotiam administered intravenously in a double dose could cause seizures within a few hours. In addition, these seizures may be referred to as early PCS.

In our opinion, one of the predisposing factors was that the damaged blood–brain barrier would permit the entrance of cefotiam into the brain parenchyma. A high concentration of the drug is more likely to cause seizures. A case report by Michenfelder et al on seizures induced by antibiotics showed a similar mechanism, wherein intraoperative intravenous penicillin administration induced early PCS in patients undergoing neurosurgery.^[7]^ Focal seizures can be evoked by topical application of penicillin to the neocortex, and generalized seizures can be produced by large doses of systemic penicillin administration. In addition, surgical manipulation of the cortex damages the blood-brain barrier, allowing easier entry of the drug into the brain. Preoperatively administered high doses of cefotiam lead to increased concentrations in the plasma and brain parenchyma. According to the pharmacokinetics of cefotiam, it is found to have a dose-dependent mechanism, and its plasma clearance decreases at higher doses due to the saturation of tubular secretion.^[12,13]^ Particularly, with renal impairment, the accumulation of toxic organic acids competes with cephalosporins for active exotransportation from the CSF to the blood, thus increasing the cephalosporin concentration in the CSF. It has also been reported that patients who experience seizures have high-dose antibiotic administration, renal impairment, or both.^[8]^

In conclusion, when a double dose of a prophylactic antibiotic drug cefotiam was administered, early PCS occurred in almost every patient. This report provides a detailed time course of early PCS after the intravenous administration of cefotiam. Furthermore, in open craniotomy surgery, prophylactic antibiotic administration must be performed carefully because intravenous antibiotics, including cefotiam, can provoke PCS.^[6,12,14]^

## Author contributions

**Conceptualization:** Dae-Lim Jee.

**Data curation:** Hyuckgoo Kim.

**Investigation:** Hyuckgoo Kim. Nawon Lee.

**Supervision:** Dae-Lim Jee. Hyuckgoo Kim.

**Writing—original draft:** Nawon Lee, Hyuckgoo Kim.

**Writing—review and editing:** Dae-Lim Jee, Hyuckgoo Kim.
